# Period doubling cascades of limit cycles in cardiac action potential models as precursors to chaotic early Afterdepolarizations

**DOI:** 10.1186/s12918-017-0422-4

**Published:** 2017-04-04

**Authors:** Philipp Kügler, M.A.K. Bulelzai, André H. Erhardt

**Affiliations:** 1 Institute of Applied Mathematics and Statistics, University of Hohenheim, Schloss 1, Stuttgart, 70599 Germany; 2 Radon Institute for Computational and Applied Mathematics, Austrian Academy of Sciences, Altenbergerstrasse 69, Linz, 4040 Austria

**Keywords:** Nonlinear dynamics, Chaos, Bifurcation theory, Cardiac action potential, Early afterdepolarizations

## Abstract

**Background:**

Early afterdepolarizations (EADs) are pathological voltage oscillations during the repolarization phase of cardiac action potentials (APs). EADs are caused by drugs, oxidative stress or ion channel disease, and they are considered as potential precursors to cardiac arrhythmias in recent attempts to redefine the cardiac drug safety paradigm. The irregular behaviour of EADs observed in experiments has been previously attributed to chaotic EAD dynamics under periodic pacing, made possible by a homoclinic bifurcation in the fast subsystem of the deterministic AP system of differential equations.

**Results:**

In this article we demonstrate that a homoclinic bifurcation in the fast subsystem of the action potential model is neither a necessary nor a sufficient condition for the genesis of chaotic EADs. We rather argue that a cascade of period doubling (PD) bifurcations of limit cycles in the full AP system paves the way to chaotic EAD dynamics across a variety of models including a) periodically paced and spontaneously active cardiomyocytes, b) periodically paced and non-active cardiomyocytes as well as c) unpaced and spontaneously active cardiomyocytes. Furthermore, our bifurcation analysis reveals that chaotic EAD dynamics may coexist in a stable manner with fully regular AP dynamics, where only the initial conditions decide which type of dynamics is displayed.

**Conclusions:**

EADs are a potential source of cardiac arrhythmias and hence are of relevance both from the viewpoint of drug cardiotoxicity testing and the treatment of cardiomyopathies. The model-independent association of chaotic EADs with period doubling cascades of limit cycles introduced in this article opens novel opportunities to study chaotic EADs by means of bifurcation control theory and inverse bifurcation analysis. Furthermore, our results may shed new light on the synchronization and propagation of chaotic EADs in homogeneous and heterogeneous multicellular and cardiac tissue preparations.

**Electronic supplementary material:**

The online version of this article (doi:10.1186/s12918-017-0422-4) contains supplementary material, which is available to authorized users.

## Background

Chaos can be defined as an aperiodic long-term behaviour in a deterministic dynamical system (either a differential equation or an iterated map/difference equation) that shows sensitive dependence on the initial conditions [1]. Though biological systems are affected by intrinsic and external stochastic noise, experimentally recorded irregular dynamics in the action potential (the characteristic membrane voltage response to a superthreshold electric stimulus) of cardiomyocytes have still been shown to be of the chaotic nature [2, 3]. More precisely, it has been observed in [2, 3] that by increasing the frequency of the stimulating current (or correspondingly by decreasing the pacing cycle length (PCL)), the 1:1 entrainment of the action potential is lost and a sequence of different m:n rhythms with alterations in the action potential duration (APD) called AP alternans is obtained before the dynamics finally become irregular. Using an iterated map

1 $$ APD_{n+1} = F(APD_{n},PCL)  $$

derived from the experimentally obtained restitution curve (APD plotted against the diastolic interval (DI)), it has been shown that, as the PCL of the stimulus is decreased, the slope of *F* at the fixed point APD^∗^ of Eq. (1) becomes progressively steeper until *∂* *F*(APD^∗^,PCL_crit_)/*∂*APD=−1 at some critical value PCL_crit_. At this flip bifurcation point PCL_crit_ the stability of the fixed point is lost and a period-2 cycle of Eq. (1) is born. The latter marks the beginning of a cascade of period-doubling bifurcations, also compare with [4], that is produced as PCL is further decreased and that finally leads to chaotic sequences of APD generated by Eq. (1). This is in accordance with the PD route to chaos in the general theory of iterated maps [1]. For later reference we emphasize that [2] studied chaotic APD variations in periodically forced cardiac pacemakers cells (i.e., they show spontaneous action potential oscillations in the absence of a stimulus), while [3] studied chaotic APD variations in periodically forced non-spontaneously active Purkinje fibre and ventricular muscle cells.

Chaotic early afterdepolarizations (EADs) are a different type of irregular cardiac action potential dynamics (different from chaotic AP alternans) that have been experimentally observed both in periodically stimulated ventricular cardiomyocytes [5] and more recently in spontaneously beating human induced pluripotent stem cell derived cardiomyocytes (hiPSC-CMs) [6–8]. EADs are abnormal voltage oscillations during the repolarization phase of the action potential, characterized by one or more periods of positive voltage slope before the normal repolarization is completed. While the irregular appearance of EADs has also been associated with stochastic activities of the ion channels (that regulate the action potential formation) in [9], the first mathematical evidence for the chaotic nature of EADs in dependence of PCL has been given in [5]. Using simulations of a deterministic differential equation model of ventricular action potential dynamics with a voltage equation

2 $$ C \frac{dV}{dt} = -\sum\limits_{ion} I_{ion} + I_{sti}(t,PCL),  $$

a restitution curve *APD*=*r*(*DI,PCL*_*crit*_), similar to the one shown in Fig. 2PP, was constructed. This curve was then used to derive Eq. (1) with *F*(*APD,PCL*)=*r*(*PCL*−*APD,PCL*) due to

3 $$ DI = PCL-APD.  $$

**Fig. 1:**
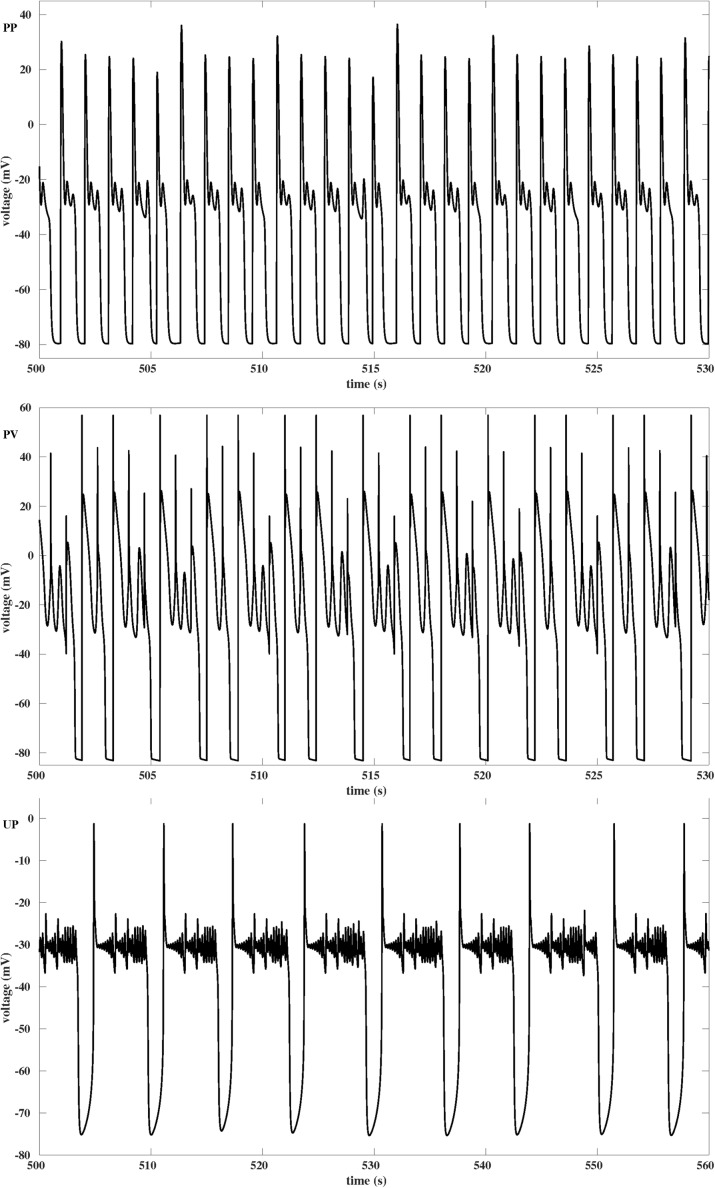
Simulation of Chaotic EADs. Numerical simulations of EADs using the deterministic AP models PP, PV and UP as outlined in the methods section. Model simulations were carried out for a time span of 2000 seconds, plots show short sections long after possible transients are gone. Positive largest Lyapunov exponents *λ* of the time series confirm the chaotic nature of the EADs. ***PP***) Chaotic EADs, *λ*=4.7*s* ^−1^, for the periodically forced pacemaker cell model PP with *G*_*K*_=0.04 mS/ cm^2^ as previously shown in [12]. For *I*_*sti*_ we chose periodic step pulses with PCL = 1.075s, step duration d = 0.002s and step amplitude A = 42 *μ* *A*/cm^2^. ***PV***) Chaotic EADs, *λ*=5.4*s* ^−1^, for the periodically paced ventricular cell model PV with *G*_*K*_=0.282 mS/ cm^2^ as previously reported in [10]. For *I*_*sti*_ we chose periodic step pulses with PCL = 0.7s, step duration d = 0.002s and step amplitude A = 30 *μ* *A*/cm^2^. As opposed to A), stimulation of the cell also takes place before full repolarization. ***UP***) Chaotic EADs, *λ*=2.7*s* ^−1^, for the unforced pacemaker cell model UP with *G*_*K*_=0.039218 mS/ cm^2^. Note that simulated chaotic EADs have previously only been published in context of periodic forcing

**Fig. 2:**
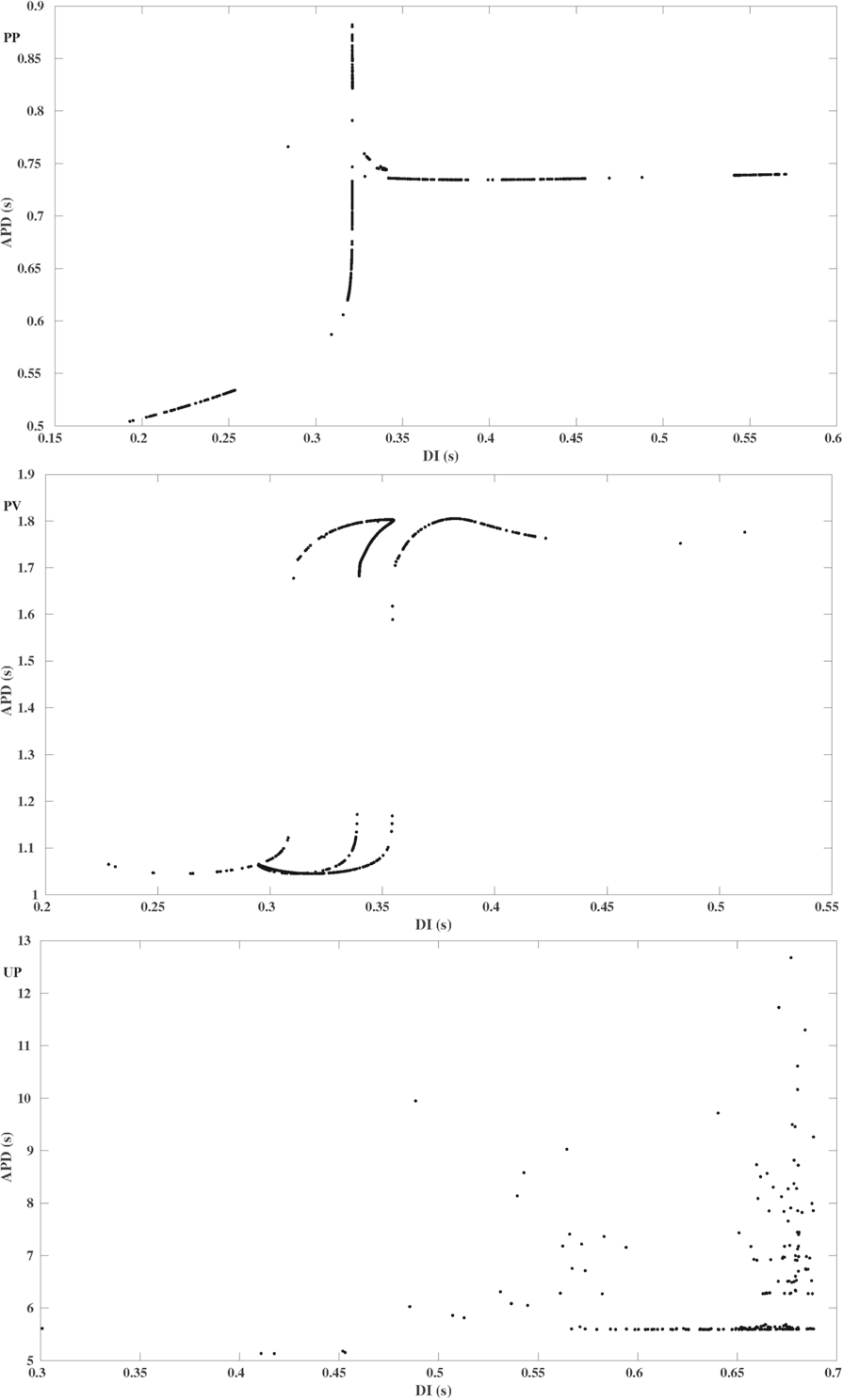
Restitution Curves obtained from Chaotic Voltage Traces. Plots of the action potential duration *APD*_*n*+1_ vs. the diastolic interval *DI*_*n*_ as obtained directly from the simulated voltage traces. ***PP***) APD restitution curve corresponding to Fig. 1PP as previously shown in [12] and similar to the APD restitution curve presented in [5]. There, the steepness of the slope of the curve has been identified as the reason for obtaining chaotic APD variations, see our discussion of the map Eq. (1). As the cell is only stimulated after full repolarization, the same restitution curve is obtained when using Eq. (3) for the determination of DI. ***PV***) APD restitution corresponding to Fig. 1PV, not previously published. As opposed to PP, the location of the data points no longer justifies to consider the relation (APD,DI) as a single-valued function. Consequently, a map Eq. (1) for the analysis of the chaotic EAD dynamics cannot be constructed. Using Eq. (3) one would obtain negative values of DI due to stimulations before full repolarization. ***UP***) APD restitution corresponding to Fig. 1UP, which even further deviates from PP than PV. Again, the scattered location of (APD,DI) data points prevents to analyze the chaotic EAD dynamics via Eq. (1). As PCL is not involved, Eq. (3) is not applicable

Finally, it has been argued in [5] that the steep positive slope of the restitution curve *r* before the peak (which translates into a steep negative slope of the map *F* at the fixed point *APD* ^∗^) proves the chaotic nature of the EAD dynamics.

In this paper, we demonstrate that a steep slope of the restitution curve *r* cannot serve as a general explanation of chaotic EAD dynamics displayed by cardiac AP models. Indeed, we will show that chaotic EAD dynamics in Eq. (2) are possible even if extracted ADP and DI data points do not form a function *r* (hence, not even admitting to speak of a slope). However, the key contribution of our paper will be novel insight into chaotic EAD dynamics gained from mathematical bifurcation studies of differential equation models of the form Eq. (2). Using a separation into fast and slow time scale variables, bifurcation analysis has been previously applied in [10] for the illumination of EADs in (a variant of) the periodically driven LR91-model [11] for ventricular cardiomyocytes. In particular, it was shown in [10] that the fast subsystem of Eq. (2) features a supercritical Hopf bifurcation from which stable limit cycles emerge until they terminate at a homoclinic bifurcation of a saddle equilibrium. Then, EAD behaviour is obtained if the model parameters are set such that the state trajectory of the full system Eq. (2) temporarily coils around the limit cycle surface spanned between the supercritical Hopf and the homoclinic bifurcations of the fast subsystem. Furthermore, the homoclinic bifurcation in the fast subsystem has been introduced in [10] as the reason for the chaotic EAD dynamics that could be obtained whenever the PCL was chosen appropriately in an EAD featuring parameter setting. Further affirmations of the statement that chaotic EAD dynamics in periodically triggered action potentials are due to a homoclinic bifurcation in the fast subsystem of Eq. (2) are given in [12–15].

Recently, we have shown in [16] that EADs may occur in action potential models Eq. (2) that do not feature a supercritical Hopf bifurcation in their fast subsystem. In this paper, we now demonstrate that a homoclinic bifurcation in the fast subsystem of Eq. (2) is neither a necessary nor a sufficient condition for obtaining chaotic EAD dynamics.

We rather argue that a PD cascade of limit cycles in the full action potential system Eq. (2) paves the way to chaotic EAD dynamics in a model-independent manner and present examples with models of a) periodically paced and spontaneously active cardiomyocytes, b) periodically paced and non-active cardiomyocytes as well as c) unpaced and spontaneously active cardiomyocytes. Furthermore, we reveal that chaotic EAD dynamics may coexist in a stable manner with regular action potential dynamics, where only the initial conditions decide about the dynamics displayed. The results of our article on chaotic EAD dynamics in single cardiomyocytes may shed new light on the synchronization of chaotic EADs [5, 17] and EAD-mediated fibrillation [18] in cardiac tissue, may open new paths for the control of cardiac chaos [19] and may be of relevance within the CiPA-initiative [20] for a new approach to preclinical drug cardiotoxicity testing with hiPSC-CMs, which considers EADs as a potential mechanism-based metric for the assessment of proarrhythmic risk.

## Methods

### Cardiac action potential models

Modelling of cardiac action potentials with systems of nonlinear ordinary differential equations (ODEs) (in which the voltage Eq. (2) is coupled to differential equations that describe the dynamics of the ion channel currents *I*_*ion*_) dates back to the work of Denis Noble [21] on both Purkinje fibre and pace-maker cells. Modern cardiac AP models for animal [22], human adult [23] and human induced pluripotent stem cell derived [24] cardiomyocytes comprise dozens of state variables and hundreds of model parameters. Typically, the only explicit time-dependence of cardiac AP models is due to the stimulating current *I*_*sti*_ such that without stimulation, i.e., *I*_*sti*_≡0, the AP model forms an autonomous ODE system. Depending on the actual equations and the particular choice of the model parameters, the autonomous ODE system may show limit cycle behaviour (then modelling spontaneous beating activity such as in pace-maker cells) or steady state behaviour (then modelling cells that show no spontaneous AP activity such as ventricular cardiomyocytes). Using inverse bifurcation analysis with sparsity promoting penalization [25, 26], models for spontaneously beating cells can be transferred into models for non-active cells and vice versa. Anyhow, the application of a periodic stimulus *I*_*sti*_ with pacing cycle length PCL leads to AP models of either periodically paced and spontaneously active or periodically paced and spontaneously non-active cells. In our study we used the following representatives for each of the three available classes of AP models. If the values of the model parameters are not explicitly mentioned in the text, they were chosen to be identical to those used in the original publications.

**AP Model PP –Periodically paced pacemaker cell**

As an example of an AP model of periodically paced and spontaneaously active cells we chose

4 $$\begin{array}{@{}rcl@{}} C \frac{dV}{dt} &=& - G_{Ca} d_{\infty}(V) f (V-E_{Ca}) - G_{K} x (V-E_{K})\\ &&+\, I_{sti}(t,PCL),  \\ \frac{df}{dt} &=& \frac{f_{\infty}(V)-f}{\tau_{f}},  \\ \frac{dx}{dt} &=& \frac{x_{\infty}(V)-x}{\tau_{x}},  \end{array} $$

as introduced in [12] and subsequently also used in [16, 27]. In particular, this ODE model of state dimension *n*=3 includes the inward calcium current

$$I_{Ca} = G_{Ca} d_{\infty}(V) f (V-E_{Ca}) $$

with the calcium channel conductance *G*_*Ca*_ and the dynamic inactivation variable *f*, as well as the outward potassium current

$$I_{K} = G_{K} x (V-E_{K}) $$

with the potassium channel conductance *G*_*K*_ and the dynamic activation variable *x*. In our discussion we used this model with the original setting *τ*_*f*_=80 and *τ*_*x*_=300 for the relaxation variables [12] and the initial conditions *y*_0_=(*V*_0_,*f*_0_,*x*_0_)=(−79.46,0.9989,0.1153).

**AP Model PV – Periodically paced ventricular cell**

As an example of an AP model of periodically paced and spontaneously non-active cells we chose

5 $$\begin{array}{@{}rcl@{}} C \frac{dV}{dt} &=& - I_{Ca}(V) - I_{K}(V) - I_{Na}(V) - I_{0}(V)\\&& +\, I_{sti}(t,PCL),  \\ \frac{dd}{dt} &=& \frac{d_{\infty}(V)-d}{\alpha \tau_{d}(V)}, \; \frac{df}{dt} = \frac{f_{\infty}(V)-f}{\beta \tau_{f}(V)}, \\ \frac{dx}{dt} &=& \frac{x_{\infty}(V)-x}{\gamma \tau_{x}(V)},\\ \frac{dh}{dt} &=& \frac{h_{\infty}(V)-h}{ \tau_{h}(V)}, \; \frac{dm}{dt} = \frac{m_{\infty}(V)-m}{ \tau_{m}(V)}, \\ \frac{dj}{dt} &=& \frac{j_{\infty}(V)-j}{ \tau_{j}(V)}. \end{array} $$

This model was previously used in [10] for the study of chaotic EADs and is a slightly modified version of the LR91-model [11] for ventricular cardiomyocytes in which the intracellular calcium now is set constant. Here, the currents depending on the dynamic gating variables *d*, *f*, *x*, *h*, *m* and *j* are

$$\begin{array}{@{}rcl@{}} I_{Ca} &=& G_{Ca} d f (V-E_{Ca}) \\ I_{K} &=& G_{K} x \bar{x}(V) (V-E_{K}) \\ I_{Na} &=& G_{Na} m^{3} h j (V-E_{Na}), \end{array} $$

while *I*_0_(*V*) summarizes those currents that are not directly driven by the dynamic gating variables. In our study we chose the relaxation parameters *α*=1, *β*=1 and *γ*=2.5 (as in Figure 4 of [10]) and the initial conditions *y*_0_=(*V*_0_,*d*_0_,*f*_0_,*x*_0_,*h*_0_,*m*_0_,*j*_0_)

6 $${} {\small{\begin{aligned} y_{0} = (-84.5286,3\cdot 10^{-6},1,0.3158,0.9832,0.0017,0.995484) \end{aligned}}}  $$

as also considered in [10].

**AP Model UP – Unpaced pacemaker cell**

As an example of an AP model of unforced and spontaneously active cells we chose Eq. (4) with the setting *τ*_*f*_=18, *τ*_*x*_=100 (from [27], see also [16]) and *I*_*sti*_≡0, and the initial conditions *y*_0_=(*V*_0_,*f*_0_,*x*_0_)=(−75.16,0.9984,0.03976).

### Numerical simulation of action potential models

For the numerical simulation of the AP models PP (paced pacemaker), PV (paced ventricular) and UP (unpaced pacemaker) we used the MATLAB [28] solver ode15s for stiff ODE systems with the relative tolerance option set to 10^−12^. All models were simulated for a time span of 2000 s. Then, the first 500 s were discarded in order to eliminate possible transient effects, such that only the remaining 1500 s of the simulations were used for the analysis.

### Numerical bifurcation analysis of AP models

Due to the nonlinearities involved, cardiac AP models Eq. (2) may show a variety of complex dynamical behaviour. Bifurcation analysis [29, 30] is the study of the dynamical repertoire and its dependence on the model parameters. Bifurcations are qualitative changes in the dynamics as model parameters are varied, and the parameter values at which they occur are referred to as bifurcation points. In the bifurcation analysis of the AP models PP, PV and UP we used the potassium channel conductance *G*_*K*_ as the primary bifurcation parameter and, for models PP and PV, the pacing cycle length *PCL* as the secondary one. The bifurcation diagrams of the AP models PP, PV and UP were obtained by means of the numerical continuation packages matcont [31] and auto-07p [32]. Continuation is the technique of following a particular solution (such as a fixed point or a limit cycle) of an autonomous system as the continuation parameter changes. One advantage of continuation is that both stable and unstable solution branches can be calculated while bifurcations of fixed points and limit cycles can be simultaneously detected. For the application of this technique to the non-autonomous models PP and PV, the latter were transformed into autonomous models of increased state dimension. To this end we introduced the two-dimensional dynamical system

7 $$\begin{array}{@{}rcl@{}} \frac{du_{1}}{dt} & = & u_{1}\left(1-u_{1}^{2}-u_{2}^{2}\right) - \frac{2 \pi}{PCL} u_{2},  \\ \frac{du_{2}}{dt} & = & u_{2}\left(1 - u_{1}^{2} - u_{2}^{2}\right) + \frac{2 \pi}{PCL} u_{1},  \end{array} $$

that admits an asymptotically stable periodic orbit [33], and appended it to Eq. (2) with the setting

8 $${} {\small{\begin{aligned} I_{sti} = \frac{A}{1+\text{exp} \left[ 5 \cdot 10^{6} \left\{ (1-u_{1}) \cos \left(\frac{d \pi}{PCL} \right) - u_{2} \sin \left(\frac{d \pi}{PCL} \right) \right\} \right] }. \end{aligned}}}  $$

Here, *A* and *d* denote the amplitude and the duration of the step pulse, see the Additional file 1 for further details.

The stable branches of the bifurcation diagrams were cross-checked and partially complemented (in case of a numerical continuation failure) by a parametric sweep, i.e., by numerical simulations of Eq. (2) that were repeated for a variety of different parameter values.

### Multiple time scale analysis of AP models

Often, cardiac AP models Eq. (2) have state variables with time derivatives of much smaller magnitude than those of other variables. Then, multiple time scale analysis [34] can be used to study the flow of the full system Eq. (2) by splitting the variables into slow and fast ones and by analyzing the corresponding slow and fast subsystems of Eq. (2). One underlying rationale is that the trajectory of the full system under certain conditions evolves along the bifurcation scaffold built by the fast subsystem in which the slow variables serve as bifurcation parameters. In particular, the multiple time scales approach was followed in [10] to discuss the genesis of EADs. More precisely, the authors first eliminated the sodium current *I*_*Na*_ from Eq. (5) (as of minor relevance for the AP repolarization phase) in order to arrive at an AP model with state dimension n=4. Then, considering the potassium gating variable *x* as much slower (and also eliminating the stimulating current *I*_*sti*_), the fast subsystem

9 $${} {{\begin{aligned} C \frac{dV}{dt} &= - G_{Ca} d f (V-E_{Ca}) - G_{K} x \bar{x}(V) (V-E_{K}) - I_{0}(V), \\ \frac{dd}{dt} &= \frac{d_{\infty}(V)-d}{\alpha \tau_{d}(V)}, \\ \frac{df}{dt} &= \frac{f_{\infty}(V)-f}{\beta \tau_{f}(V)} \end{aligned}}}  $$

was derived in which *x* acts as a model parameter. Finally, a bifurcation analysis of Eq. (9) for the setting *α*=0.1 and *β*=1.1 and with *x* as the continuation parameter revealed that Eq. (9) features a supercritical Hopf bifurcation followed by a homoclinic bifurcation to a saddle fixed point. While the Hopf bifurcation was considered in [10] to be necessary for the genesis of EADs in the full system Eq. (5), the homoclinic bifurcation was introduced as the reason for the occurence of chaotic EAD dynamics in Eq. (5) under an appropriate periodic stimulation.

While, with respect to chaotic EADs, our paper suggests to rather explore the full AP dynamics of Eq. (2) than fast subsystems, we also studied for the sake of comparison the fast subsystem

10 $$\begin{array}{@{}rcl@{}} C \frac{dV}{dt} &=& - G_{Ca} d_{\infty}(V) f (V-E_{Ca}) - G_{K} x (V-E_{K}),\\ \frac{df}{dt} &=& \frac{f_{\infty}(V)-f}{\tau_{f}}  \end{array} $$

of the cardiac AP models PP and UP, then again with the slow variable *x* as the bifurcation parameter. The submodel Eq. (10) was previously used in [16] in order to demonstrate that a supercritical Hopf bifurcation in a fast subsystem is actually not necessary for EAD genesis.

### Calculation of Lyapunov exponents

Cardiac AP models Eq. (2) may feature both regular and chaotic EAD dynamics. In order to test whether a given trajectory is actually chaotic, we calculated the largest Lyapunov exponent *λ* from the simulated voltage time series data using the TISEAN package [35]. The central idea is to consider a trajectory on the presumably chaotic attractor and to pick a point $\mathbf {s}_{n_{1}}$ in the state space. Then, a second point $\phantom {\dot {i}\!}\mathbf {s}_{n_{2}}$ in close distance $\delta _{0} = \|\mathbf {s}_{n_{1}}-\mathbf {s}_{n_{2}}\|\phantom {\dot {i}\!}$ is chosen, and the separation of the two trajectories over time is measured in terms of

$$\| \mathbf{s}_{n_{1} + \Delta n}-\mathbf{s}_{n_{2} + \Delta n} \| \approx \delta_{0} e^{\lambda \Delta n}. $$

A positive value of *λ* corresponds with an exponentially fast growth of the initial perturbation *δ*_0_ and means that the respective trajectory is chaotic.

### Calculation of restitution curves

As considered in [5] and [12] as an alternative to the S1-S2-protocol, we derived the restitution curves directly from the simulated voltage traces. To this end, all time points at which the voltage crosses the threshold value *V*_*th*_ were recorded during the simulations using the MATLAB event option of the ODE solver. Action potential durations APDs were then calculated as the time spans during which the voltage lies above *V*_*th*_, while diastolic intervals DIs were constructed as the time spans with voltage values below *V*_*th*_. Finally, pairs of *DI* and subsequent *APD* were built and plotted as *APD*_*n*+1_ vs. *DI*_*n*_. Our motivation for the use of this direct method was its straightforward applicability to both paced and spontaneaously active models without the need of introducing perturbations to the latter. For the sake of completeness, we also applied the S1-S2-protocol, see the Additional file 2.

## Results

Simulations of chaotic EADs via the numerical integration of Eq. (2) have so far only been reported [5, 10, 12] for the case of a periodic stimulation by an external current *I*_*sti*_, see Figs. 1PP and 1PV for examples. However, studying drug induced EADs in spontaneously beating human induced pluripotent stem cell derived cardiomyocytes, we found that chaotic EADs may also form in simulations of AP models without periodic stimulation, see Fig. 1UP. Note that a comparable high number of small oscillations during EAD-like activity has been experimentally observed in [36]. As the occurence of chaotic EADs of the type shown in Fig. 1PP (i.e., the AP is only triggered by the external current after full repolarization) has been attributed in [5, 12] to the steep slope of the APD restitution curve, see Fig. 2PP, we first wondered if this argument may also apply to chaotic EADs of the types shown in Figs. 1PV (external stimulation also before full repolarization) and 1UP (no external stimulation at all). Having derived the corresponding APD restitution curves, see Figs. 2PV and 2UP, we realized that they strongly deviate from their previously published counterparts as exemplified in Fig. 2PP. In particular, due to the lack of continuity and differentiability properties the “APD restitution curves” of Figs. 2PV and 2UP do not allow to define maps Eq. (1) for the iteration of APD and hence do not contribute to the understanding of the chaotic EAD types shown in Figs. 1PV and 1UP.

**Fig. 3:**
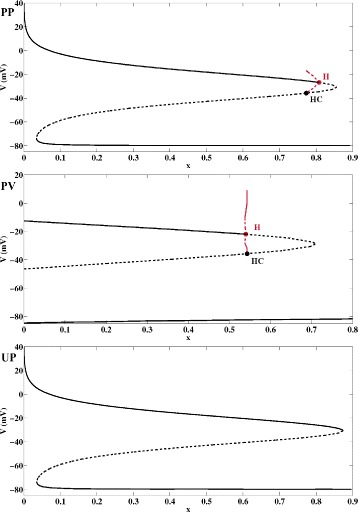
Bifurcation Diagrams of Fast AP Subsystems. ***PP***) Bifurcation diagram of the fast subsystem Eq. (10) of model PP with the potassium gating variable *x* as continuation parameter. The solid and dashed black lines denote stable and unstable fixed points of Eq. (10). At the subcritical Hopf bifurcation H, unstable limit cycles emerge that subsequently terminate at a saddle-homoclinic bifurcation HC. The red dashed lines show the maximum and minimum voltage values of the unstable limit cycles. ***PV***) Bifurcation diagram of the fast subsystem Eq. (9) (appended by the equations for *h*, *m* and *j* from Eq. (5)) of model PV, *α*=1 and *β*=1, with the potassium gating variable *x* as continuation parameter. The solid and dashed black lines denote stable and unstable fixed points. At the subcritical Hopf bifurcation H, unstable limit cycles are born that turn into stable ones at a saddle node of cycles bifurcation before they terminate at a saddle-homoclinic bifurcation HC. The red dashed lines show the maximum and minimum voltage values of unstable limit cycles, the red solid lines show the extreme voltage values of stable limit cycles. ***UP***) Bifurcation diagram of the fast subsystem Eq. (10) of model UP with the potassium gating variable *x* as continuation parameter. The solid and dashed black lines denote stable and unstable fixed points of Eq. (10) that annihilate each other at saddle-node bifurcations. As opposed to ***PP***) and ***PV***), neither a Hopf nor a homoclinic bifurcation does exist, and neither stable nor unstable limit cycles are detected. Still, model UP features chaotic EAD dynamics, see Fig. 1UP

In our attempt to find a common explanation for the chaotic EAD dynamics observed in Figs. 1PP, 1PV and 1UP, we next focused on the hypothesis featured in [10, 12–15] according to which chaotic EADs have their source in a saddle-homoclinic bifurcation in the fast AP subdynamics. While in [10] the homoclinic bifurcation occurs after a supercritical Hopf bifurcation, our analysis of the fast subystems of models PP and PV shows that in these examples the homoclinic bifurcation is rather accompanied by a subcritical Hopf bifurcation of which only unstable limit cycles emerge, see Figs. 3PP and 3PV. However, the bifurcation analysis of the fast subsystem Eq. (10) of the AP model UP reveals that in this case a homoclinic bifurcation is not involved at all, see Fig. 3UP. It has been previously shown in [16], that the reason for the occurence of EADs in model UP is a saddle-focus fixed point whose unstable manifold causes small scale oscillations of growing amplitudes. Since the AP model UP still features chaotic EADs, see Fig. 1UP, it is demonstrated that a homoclinic bifurcation in the fast AP subdynamics is in fact not a necessary condition for the occurence of chaotic EADs.

**Fig. 4:**
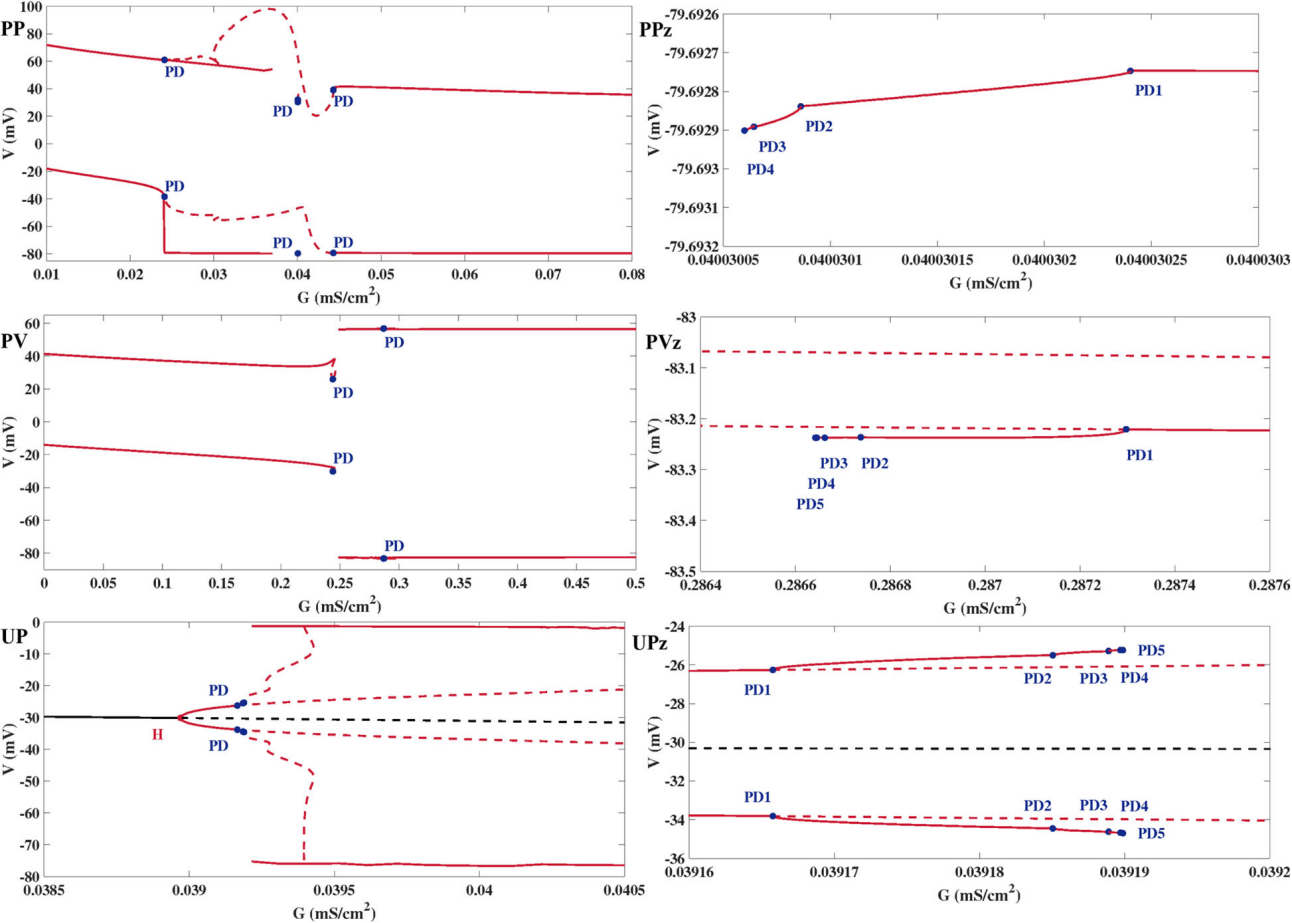
Bifurcation Diagrams of Full AP Systems. The red dashed lines show the maximum and minimum voltage values of the unstable limit cycles of the full AP systems that were detected, the red solid lines correspond to stable limit cycles. In ***PP***), ***PV***), ***UP***), the blue markers depict the beginning of a cascade of period doubling bifurcations of stable limit cycles, in ***PPz***), ***PVz***), ***UPz***) the blue markers show the PD bifurcations of a particular cascade in close vicinity to the *G*_*K*_ value resulting in the chaotic EAD dynamics of Fig. 1. ***PP***) Bifurcation diagram of model PP with the potassium conductance *G*_*K*_ as continuation parameter (*I*_*sti*_ chosen as for Fig. 1PP). ***PV***) Bifurcation diagram of model PV with the potassium conductance *G*_*K*_ as continuation parameter (*I*_*sti*_ chosen as for Fig. 1PV). ***UP***) Bifurcation diagram of model UP with the potassium conductance *G*_*K*_ as continuation parameter. The solid and dashed black lines denote stable and unstable fixed points of model PP. At the supercritical Hopf bifurcation, stable limit cycles of small amplitudes emerge that go through a cascade of PD bifurcations, depicted by the blue markers, before they turn into limit cycles of large amplitudes that correspond to periodic EAD dynamics. In between lies an area that features both periodic and chaotic dynamics, including chaotic EAD dynamics as shown in Fig. 1UP. ***PPz***) Zoom into the PD area of ***PP***), ***PVz***) zoom into the PD area of ***PV***), and ***UPz***) zoom into the PD area of ***UP***), all in neighborhood of the *G*_*K*_ values corresponding to the chaotic EAD traces shown in Figures 1PP-1UP. This suggests that the PD points numerically detected are part of an infinite PD cascade leading to chaotic EADs

**Fig. 5:**
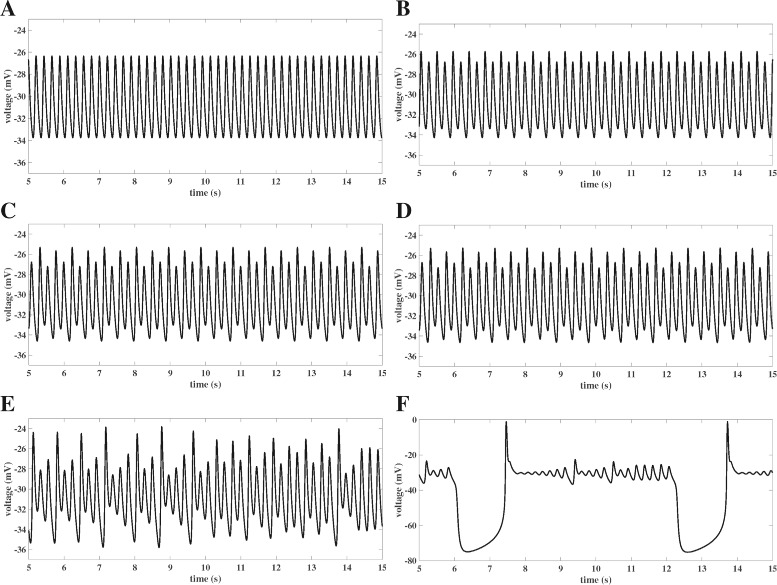
Period Doubling Route to Chaotic EADs of Model UP. Evolution of voltage trajectories of model UP as the channel conductance *G*_*K*_ is increased along the period doubling cascade PD1, PD2, PD3,... **A**) Single period with *G*_*K*_=0.039155 mS/ cm^2^ and $\protect \phantom {\dot {i}\!}G_{K} < G_{K_{1}}$. **B**) Double period with *G*_*K*_=0.039175 mS/ cm^2^ and $\protect \phantom {\dot {i}\!}G_{K_{1}} < G_{K} < G_{K_{2}}$. **C**) Fourfold period with *G*_*K*_=0.039188 and $\protect \phantom {\dot {i}\!}G_{K_{2}} < G_{K} < G_{K_{3}}$. **D**) Eightfold period with *G*_*K*_=0.039189 mS/ cm^2^ and $G_{K_{3}} < G_{K} < G_{K_{4}}$. **E**) Chaotic motion of failed repolarization with *G*_*K*_=0.039205 mS/ cm^2^. **F**) Chaotic EADs behaviour with *G*_*K*_=0.039218 mS/ cm^2^

Neither the EAD-theory based on the steepness of AP restitution curves nor the EAD-theory based on homoclinic bifurcations in fast AP subsystems can attribute the chaotic EAD dynamics of Figs. 1PP, 1PV and 1UP to a common cause. In particular, none of these theories can shed light on the chaotic EAD dynamics of model UP, as it neither admits a steep AP restitution curve nor a homoclinic bifurcation in the fast subsystem. For gaining insight, we hence decided to perform a bifurcation analysis of the full AP system of the model UP with the potassium channel conductance *G*_*K*_ as the continuation parameter. While in principle also other model parameters could be chosen for the continuation, the choice of *G*_*K*_ is motivated by the strong focus of the established drug safety guidelines on potencies to block potassium currents. The corresponding bifurcation diagram of Fig. 4UP shows that, starting from an area of low *G*_*K*_ values that do not admit spontaneous oscillatory activity but only attraction towards fixed points, stable limit cycles of comparatively small amplitudes emerge from a supercritical Hopf point as the value of *G*_*K*_ is increased. At the period doubling point PD1, the single-period oscillation loses its stability and splits into stable double-period oscillations. As *G*_*K*_ is further increased, further period doublings PD2, PD3, PD4 and PD5 are numerically detected. Figure 5 illustrates corresponding periodic trajectories with single, double, fourfold and eightfold period before the motion becomes chaotic. Furthermore, the chaotic nature is also present after the transition from the small amplitude motion with a failed repolarization to the large amplitude motion of AP type, with one representative given by the chaotic EAD dynamics displayed in Fig. 1UP, before the AP type motion turns into periodic EAD dynamics (followed by, not shown, periodic AP dynamics and finally an attraction towards a steady state after another Hopf bifurcation). For projections of the trajectories onto the *V*-*x*-plane of the state space, see the Additional file 3.

Having associated the chaotic EAD dynamics of the model UP with the period-doubling route to chaos in ODE systems [30], we wondered if this phenomenon, numerically and experimentally observed in many other biological and physical systems including neuronal activity [37, 38], may also underlie the chaotic EAD dynamics in the non-autonomous models PP and PV. Transforming the latter into autonomous systems using Eqs. (7) and (8), we hence performed a numerical bifurcation analysis again with *G*_*K*_ as the continuation parameter. The resulting bifurcation diagrams, displayed in Figs. 4PP and 4PV, reveal that also in the case of model PP and PV, the value of *G*_*K*_ corresponding to the chaotic EAD dynamics shown in Fig. 1 in fact lies in close vicinity to a cascade of period doubling bifurcations. For illustrations of the corresponding periodic trajectories we refer to the Additional file 4.

## Discussion

Chaotic EAD dynamics in AP models Eq. (2) have been previously observed in the case of periodic pacing [5, 10, 12] but have been attributed to either the steepness of APD restitution curves or the existence of homoclinic bifurcations in fast AP subdynamics. While none of these results is able to explain the chaotic EAD dynamics observed in the unforced AP model UP, our study suggests the existence of a cascade of period doubling bifurcations of limit cycles as a model-independent explanation for chaotic EAD dynamics both in forced and unforced cardiac AP systems of the ODE type. We emphasize that period doubling bifurcations, though then observed in iterated APD maps Eq. (1) rather than in mechanistic cardiac AP models Eq. (2), have so far only been linked with chaotic APD alternans [2–4] (which constitutes a type of cardiac arrhythmia that is different from EADs).

The results of this study were obtained by means of bifurcation analysis applied to AP models Eq. (2) based on numerical continuation using the software packages matcont [31] and auto-07p [32]. In the context of EADs-research, numerical continuation was previously only applied to fast subsystems of Eq. (2), see [10, 16]. Though the analysis of fast subsystems can illuminate EAD generating mechanisms during a single AP [10, 16], its capability to study the occurence of EADs over a time span covering several APs may be limited as the conditions for a separation into fast and slow state variables [34] may not be met in the long term. In that regard, the proper consideration of the stimulating current in Eq. (9) or Eq. (10), which certainly constitutes a very fast component of the system, might be one hurdle to be taken. In contrast, the advantage of studying the dynamical behaviour by a bifurcation analysis of the full AP model Eq. (2) is that its long term behaviour defined by stable and chaotic attractors can be captured and tracked as model parameters are continuously varied. Besides of the detection of bifurcations such as Hopf, saddle node of cycles, homoclinic or period doubling bifurcations, another benefit of bifurcation analysis with numerical continuation is that unstable (i.e., non-attracting) dynamical structures of Eq. (2) can be revealed. While the interpretation of the unstable limit cycles illustrated in Fig. 4 goes beyond the scope of this study, the relevance of unstable structures in the context of AP modelling is highlighted in [39], in which the existence of an unstable chaotic invariant set suggests that the excitability of a membrane to fire an AP may be more complex than a smooth hypersurface that divides subthreshold and suprathreshold membrane potentials.

The transition between two chaotic states (chaotic low amplitude dynamics of failed repolarization and chaotic EAD dynamics) observed in the model UP, see Figs. 4 and 5, is reminiscent of the transition between chaotic spiking and chaotic bursting [37] in an unforced Hindmarsh–Rose model of neuronal activity. Still, a difference is the sharp change in amplitude of the neighboring stable limit cycles in Fig. 4UP as opposed to the comparatively same levels of amplitudes in neuronal spiking and bursting reported in [37]. Such sharp transitions in stable limit cycle amplitudes as observed in the unforced model UP are also evident in the bifurcation diagrams of the periodically forced cardiac AP models PP and PV, see Figs. 4PP and 4PV. However, the chaotic EAD traces displayed in Figs. 1PP and 1PV are obtained with parameter values of *G*_*K*_ that lie far away from these transitions but rather close to different cascades of period doubling bifurcations. A common feature of the unforced and the periodically forced models of this study is that the corresponding PD cascades are all of the supercritical type and seem to obey Feigenbaum’s law [30]

11 $$ {\lim}_{n\to \infty} \frac{G_{K_{n+1}}-G_{K_{n}}}{G_{K_{n}}-G_{K_{n-1}}} = 0.214169...,  $$

where $G_{K_{n}}$ is the parameter value of *G*_*K*_ corresponding to the *n*-period doubling *PD*_*n*_.

**Fig. 6:**
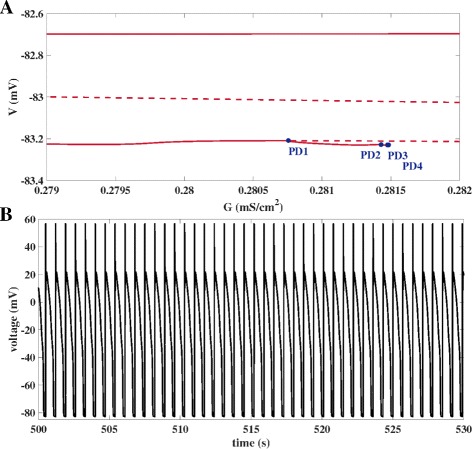
Coexistence of Chaotic EADs and Normal Periodic AP Dynamics. **A** Zoom into the PD cascade area of Fig. 4b, but now also depicting a solid red line at *V*≈−82.7 mV that represents the minimum voltage values of stable limit cycles corresponding to normal periodic AP behaviour. **B** Display of the normal periodic AP orbit obtained with the same parameter setting as for the chaotic EAD dynamics of Fig. 1PV but with the different initial condition Eq. (12)

A further exploration of the bifurcation diagram of the model PV reveals that the PD cascade is accompanied by a stable branch of limit cycles, indicated by the solid line in Fig. 6 a at *V*≈−82.7 mV. This branch corresponds to limit cycles of periodically driven regular action potentials as displayed in Fig. 6 b and demonstrates the coexistence of regular AP dynamics with the EAD affected limit cycles of the PD cascade for a certain range of potassium channel conductances *G*_*K*_. Consequently, regular AP dynamics may also coexist with chaotic EAD dynamics if *G*_*K*_ is chosen beyond the cascade-limit of periodic behaviour, see Figs 1PV and 6b which are obtained with the same value of *G*_*K*_ and identical periodic forcing but with the two different initial conditions Eq. (6) and

12 $${} {\small{\begin{aligned} y_{0} = (-82.598,0.9742,0.0023,0.9803,0.0035,0.9706,0.4809). \end{aligned}}}  $$

This is similar to the coexistence of periodic spiking and chaotic bursting reported for a non-forced spontaneously active neuron model in [40], then attributed to a bifurcation of a saddle-node periodic orbit. Hence, even though the fast subsystem of model PV features a homoclinic bifurcation, the latter is not sufficient for the occurence of chaotic EAD dynamics as in addition to the model parameters also the initial conditions need to be properly set. This coexistence is of relevance both for numerical and experimental studies of cardiac action potentials which typically are based on the assumption that after a sufficiently long transient the (one and only) “steady state” periodic behaviour is observed independently of the pacing history.

**Fig. 7:**
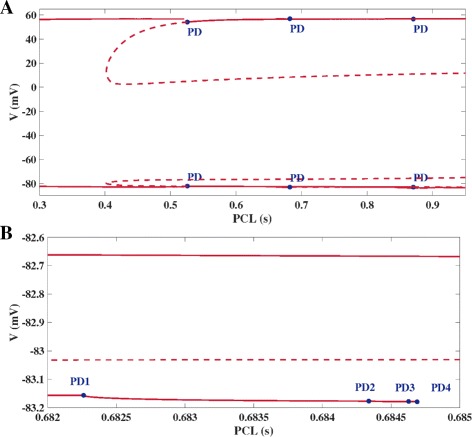
Bifurcation Diagram of Full AP System with PCL as Continuation Parameter. **A** Bifurcation diagram of model PV with the pacing cycling length PCL as the continuation parameter. Here, the channel conductance *G*_*K*_, the current step amplitude A and the step duration d were chosen as for Fig. 1PV. The red dashed and solid lines show the maximum and minimum voltage values of unstable and stable limit cycles. Blue markers depict several PD bifurcations of limit cycles spread across a wide range of PCL values. **B** Zoom into the PD area of A) in neighborhood of the PCL value corresponding to the chaotic EAD trace shown in Fig. 1PV. Again, the detection of serveral PD bifurcations that seem to obey Eq. (11) suggests that the period doubling route to chaotic EADs is on hand. The upper solid branch depicts the minimum voltage value of stable limit cycles that correspond to regular periodic AP orbits, once again demonstrating the coexistence of chaotic EAD dynamics and periodic AP dynamics in model PV

Other than channel conductances such as *G*_*K*_, the dependency of cardiac AP dynamics on the pacing cycle length PCL of the stimulating current *I*_*sti*_ is of high relevance for studies of cardioelectrophysiology. Typically, the action potential duration APD is derived from voltage traces that are simulated or experimentally recorded for a discrete sequence of different values of PCL, see [23]. Our transformation of the periodically forced and hence non-autonomous AP model Eq. (2) into an autonomous AP model of extended state based on Eqs. (7), (8) offers a complementary method for studying the impact of PCL on the dynamic repertoire of the cardiac AP model. In particular, the numerical bifurcation analysis based on a limit cycle continuation allows for a continuous PCL screening and ensures that critical PCL points or intervals are not missed as they possibly would be in case of only a discrete PCL sampling. As an example, Fig. 7 shows corresponding bifurcation diagrams for the model PV with PCL as the continuation parameter. As with the continuation of *G*_*K*_, both the period doubling route to chaos and the coexistence of two stable periodic orbits for one and the same parameter value can be detected. Furthermore, the spread of additional PD bifurcations over a wide range of PCL suggests that the PD-route to EAD chaos is a non-local phenomenon in periodically driven AP models, which is in accordance with the observation of APD chaos over a wide range of PCL in the parametric sweep studies of APD [5, 12].

We end this section with some limitations of our study. Our study focuses on the generation of chaotic EADs in ODE models Eq. (2) that represent the behaviour of single cardiomyocytes. Clearly, our work needs to be extended to PDE models of cardio-electrophysiology in order to take into account the coupling between cells and spatial AP wave propagation. Furthermore, we have not incorporated stochastic effects which also may have an impact on the bifurcation repertoire of dynamical systems. Finally, the discontinuities in the bifurcation diagrams due to a failure of the numerical continuation and the unraveled unstable limit cycle branches require further analysis to extract the full information about the dynamical repertoire of Eq. (2). Note, however, that our bifurcation approach led to the discovery of chaotic EADs in unforced cardiac AP models and furthermore offers an explanation via the PD route to chaos then also uniformly applicable to chaotic EADs in forced cardiac AP models.

## Conclusions

EADs are pathological voltage oscillations during the repolarization phase of the AP of cardiomyocytes and are considered as potential triggers of cardiac arrhythmias in context of both ion channel diseases and drug cardiotoxicity testing. In this study, we have contributed to the theory of EAD dynamics by attributing their chaotic appearance to cascades of period doubling bifurcations of limit cycles in deterministic AP models. As demonstrated in this article, the detection of PD cascades via the numerical continuation of limit cycles is possible both for paced and unforced cardiac AP models and serves as a strong indicator of chaotic EAD dynamics that then take place in immediate vicinity in the parameter space. Hence, the automatically executable detection of PD cascades in the full AP dynamics defines a parameter-to-output map *F*:*p*→*PD*(*p*) which might allow to formulate and solve associated inverse bifurcation problems [25] that address the avoidance or the control [19] of chaotic EAD dynamics in cardiomyocytes. Furthermore, PD cascades might serve as classifiers of proarrhythmicity that provide improved risk prediction in comparison with purely simulation based and unspecific markers such as APD90 or AP upstroke velocity. Also, the incorporation of drug-ion channel interaction models [41] into Eq. (2) such as, e.g., simple pore block

$$G = \frac{1}{1+\frac{D}{IC_{50}}} $$

would allow to conduct the bifurcation analysis directly with respect to drug concentration D or IC50 of the ion channels of interest. In that regard, the findings of this study might be of relevance for the currently unfolding CIPA initiative to redefine the drug safety paradigm [20]. Though the latter considers mathematical modelling and simulation of cardiac APs as one of its three pillars (next to ion channel studies and experiments with hiPSC-CMs), it seems to so far ignore the potential of bifurcation analysis for the illumination of arrhythmic and chaotic behaviour in dynamical systems.

## Additional files

Additional file 1 Transformation of Periodically Driven AP Models into Autonomous Form. (PDF 315 kb)

Additional file 2 Restitution Curves Computed via S1-S2-Stimulation. (PDF 170 kb)

Additional file 3 Period Doubling Route to Chaos of Model UP. (PDF 391 kb)

Additional file 4 Illustration of Period Doubling Route to Chaotic EADs in Models PP and PV. (PDF 1421 kb)

## Abbreviations

AP: Action potential
APD: Action potential duration
CiPA: Comprehensive In-vitro proarrhythmic assay
DI: Diastolic interval
EAD: Eearly afterdepolarization
hiPSC-CM: Human induced pluripotent stem cell derived cardiomyocyte
ODE: Ordinary differential equation
PD: Period doubling
PCL: Pacing cycle length
PP: Paced pacemaker
PV: Paced ventricular
UP: Unpaced pacemaker
