# Supplementary material for: Period doubling cascades of limit cycles in cardiac action potential models as precursors to chaotic early Afterdepolarizations
Source: BMC Syst Biol. 2017 Apr 4;11:42. doi: 10.1186/s12918-017-0422-4 (PMC5379775; doi:10.1186/s12918-017-0422-4)
Supplement: Supplementary file 1 — Transformation of Periodically Driven AP Models into Autonomous Form. (PDF 315 kb) [file 12918_2017_422_MOESM1_ESM.pdf]

Additional File 1

Transformation of Periodically Driven AP Models into Autonomous Form

The periodic stimulating current I_{sti}, typically in the form of periodic step pulses of amplitude A and duration d with periodic cycling length PCL, renders cardiac AP models that in first place are non-autonomous. The explicit time-dependence can be eliminated by expanding the AP model by the two-dimensional dynamical system

$$\begin{aligned} \frac{du_1}{dt} &= u_1(1 - u_1^2 - u_2^2) - \frac{2\pi}{PCL}u_2, \\ \frac{du_2}{dt} &= u_2(1 - u_1^1 - u_2^2) + \frac{2\pi}{PCL}u_1 \end{aligned} \tag{1}$$

with $u(0) = (1, 0)$ and by setting

$$I_{sti} = \frac{A}{1 + \exp\left[5 \cdot 10^6 \left\{(1 - u_1)\cos\left(\frac{d\pi}{PCL}\right) - u_2 \sin\left(\frac{d\pi}{PCL}\right)\right\}\right]}. \tag{2}$$

Figure 1 gives one example used in our study. That way, a periodically forced

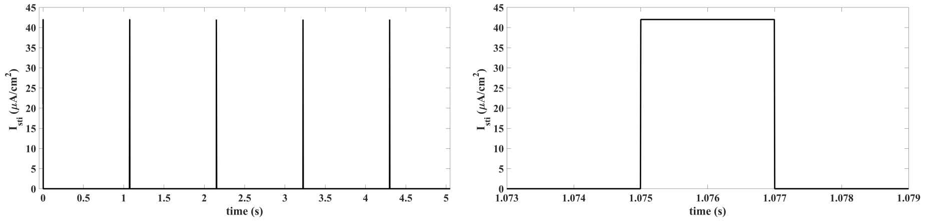

Figure 1: **Generation of periodic step pulses by solving** (1) **and using** (2)**.** Example with $PCL = 1.075$ s, $A = 42$ $\mu A/\mathrm{cm}^2$ and $d = 0.002$ as used for the analysis of the cardiac AP model PP. A) Periodic sequence of six step pulses. B) Zoom onto one of the step pulses.

cardiac AP model becomes amenable to bifurcation analysis by numerical continuation, in which in particular also the pacing cycle length PCL can be chosen as bifurcation parameter.
