# Supplementary material for: Period doubling cascades of limit cycles in cardiac action potential models as precursors to chaotic early Afterdepolarizations
Source: BMC Syst Biol. 2017 Apr 4;11:42. doi: 10.1186/s12918-017-0422-4 (PMC5379775; doi:10.1186/s12918-017-0422-4)
Supplement: Supplementary file 2 — Restitution Curves Computed via S1-S2-Stimulation. (PDF 170 kb) [file 12918_2017_422_MOESM2_ESM.pdf]

Additional File 2

Restitution Curves Computed via S1-S2-Stimulation

In the main text, the restitution curves were derived directly from the chaotic voltage traces affected by EADs. As an alternative, we also applied the S1-S2-protocol for the construction of the restitution curves. For this purpose, the models PP and PV were paced for a period of 500 seconds in order to eliminate any transient effects, the periodic stimulating currents I_{sti} were chosen as for the direct method. Then, a stimulus S2 of the same strength was applied after a time interval DI in distance from the last repolarization and the duration of the triggered action potential was measured. Repeating this procedure for various values of DI, the restitution curve APD_{n+1} vs. DI_n was constructed. In case of model UP, we simulated 500 seconds of spontaneously beating activity before introducing a stimulus S2, a cross-check with a preceding S1-pacing did not yield different outcomes. Figure 1 shows the corresponding restitution curves. The striking qualitative difference between the three models once more suggests that steepness of the restitution curve, which again is only observable for model PP, cannot serve as a universal mechanistic explanation for the onset of chaotic EADs in cardiac action potential models. While the restitution curves obtained directly from the time series data and those obtained by means of the S1-S2-protocol qualitatively conincide in case of model PP, they strongly differ in case of models PV and UP. In contrast to model PP, the chaotic EADs in models PV and UP are obtained by stimulations that in parts take place before full repolarization or solely by spontaneous activity, respectively. Hence, only in case of model PP the two methods yield a comparable outcome as only there the chaotic EADs are obtained by stimulations that exclusively take place after full repolarization. A further investigation of the dependence of the restitution curves on the methods they are computed with is beyond the scope of the manuscript and will be the subject of future studies.

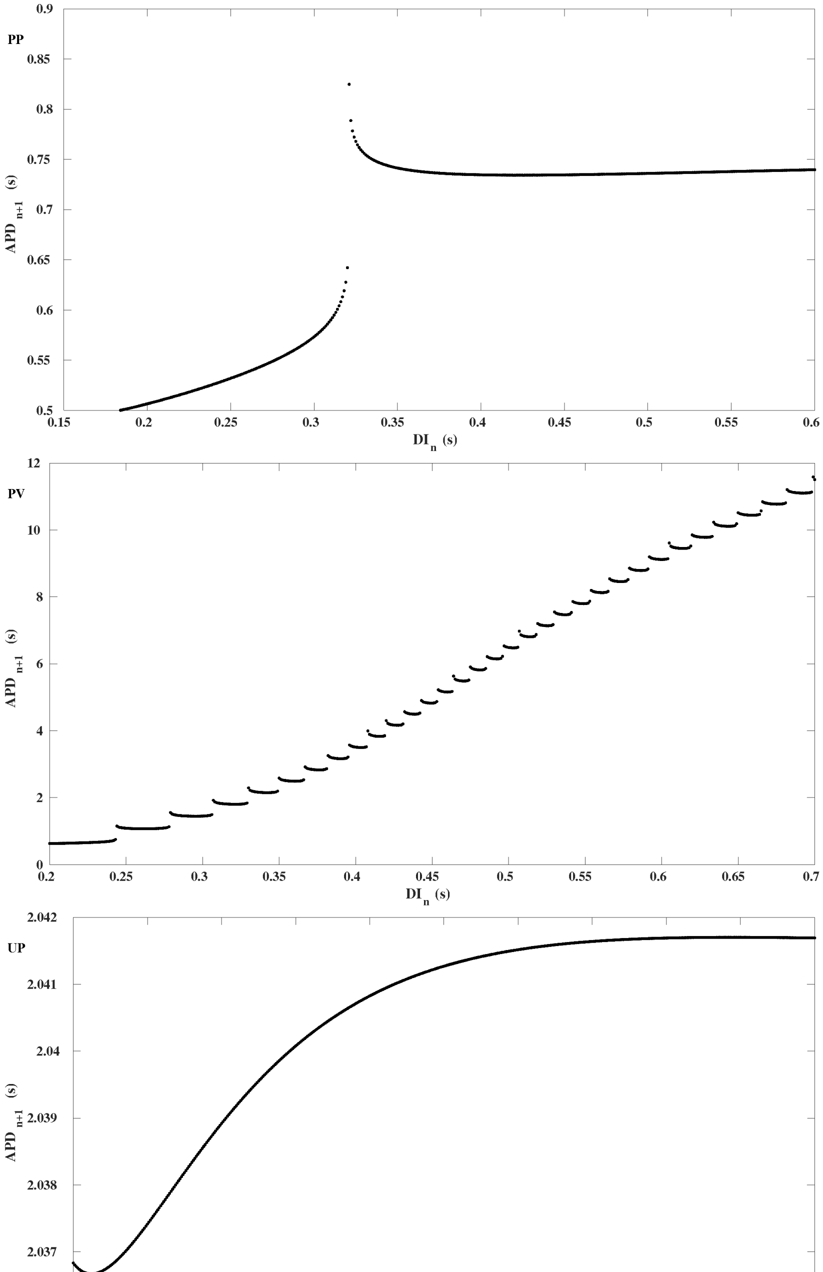

Figure 1: **Restitution Curves obtained by S1-S2-Stimulation** Display of the restitution curves for models PP, PV and UP as obtained via the S1-S2-protocol. As with the direct calculation method, steepness of restitution can only be observed in case of model PP. The discontinuities in case of model PV correspond to increases in the integer numbers of small oscillations during EAD-like activity.
