# Supplementary material for: Period doubling cascades of limit cycles in cardiac action potential models as precursors to chaotic early Afterdepolarizations
Source: BMC Syst Biol. 2017 Apr 4;11:42. doi: 10.1186/s12918-017-0422-4 (PMC5379775; doi:10.1186/s12918-017-0422-4)
Supplement: Supplementary file 3 — Period Doubling Route to Chaos of Model UP. (PDF 391 kb) [file 12918_2017_422_MOESM3_ESM.pdf]

# Additional File 3

## Period Doubling Route to Chaos of Model UP

In the main text, the period doubling route to chaos of model UP was illustrated by means of plots of the corresponding voltage time series, see Figure 5 of the manuscript. In Figure 1, we show supporting projections of the trajectories onto

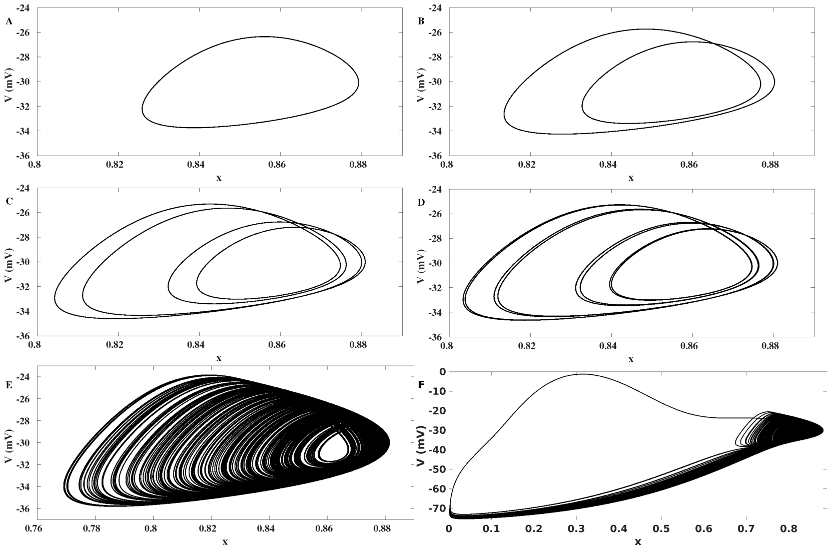


Figure 1: **Period Doubling Route to Chaotic EADs of Model UP.** Projections of the trajectories onto the $V$-$x$-plane of the state space for different values of the channel conductance $G_K$ along the period doubling cascade PD1, PD2, PD3, ... A) Single period with $G_K = 0.039155$ mS/cm$^2$ and $G_K < G_{K_1}$. B) Double period with $G_K = 0.039175$ mS/cm$^2$ and $G_{K_1} < G_K < G_{K_2}$. C) Fourfold period with $G_K = 0.039188$ and $G_{K_2} < G_K < G_{K_3}$. D) Eightfold period with $G_K = 0.039189$ mS/cm$^2$ and $G_{K_3} < G_K < G_{K_4}$. E) Chaotic motion of failed repolarization with $G_K = 0.039205$ mS/cm$^2$. F) Chaotic EADs behaviour with $G_K = 0.039218$ mS/cm$^2$.

the $V$-$x$-plane of the state space. In particular, the period doubling from the fourfold to the eightfold period becomes clearly visible that way.

Figure 2 displays the action potential durations of model UP that are obtained if the parameter $G_K$ is further increased beyond the area of the PD cascade. Chaotic behaviour is indicated by multiple values of $APD$, in accordance with Figure 4UP of the manuscript, no other parameter areas of relevance for chaotic action potential behaviour are detected.

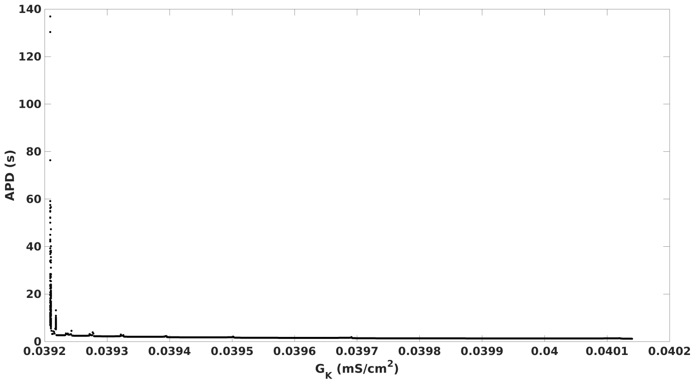


Figure 2: **Action Potential Duration vs. Channel Conductance** $G_K$**.** For parameter values larger than $G_K = 0.039218$, $APD$ is a single valued function of $G_K$ which corresponds to periodic system behaviour.
