# Supplementary material for: Period doubling cascades of limit cycles in cardiac action potential models as precursors to chaotic early Afterdepolarizations
Source: BMC Syst Biol. 2017 Apr 4;11:42. doi: 10.1186/s12918-017-0422-4 (PMC5379775; doi:10.1186/s12918-017-0422-4)
Supplement: Supplementary file 4 — Illustration of Period Doubling Route to Chaotic EADs in Models PP and PV. (PDF 1421 kb) [file 12918_2017_422_MOESM4_ESM.pdf]

Additional File 4

Illustration of Period Doubling Route to Chaotic EADs in Models PP and PV

In support of Figure 4 of the main text, the following Figures 1 and 2 display the voltage traces of the models PP and PV corresponding to the period doubling route to chaotic EADs.

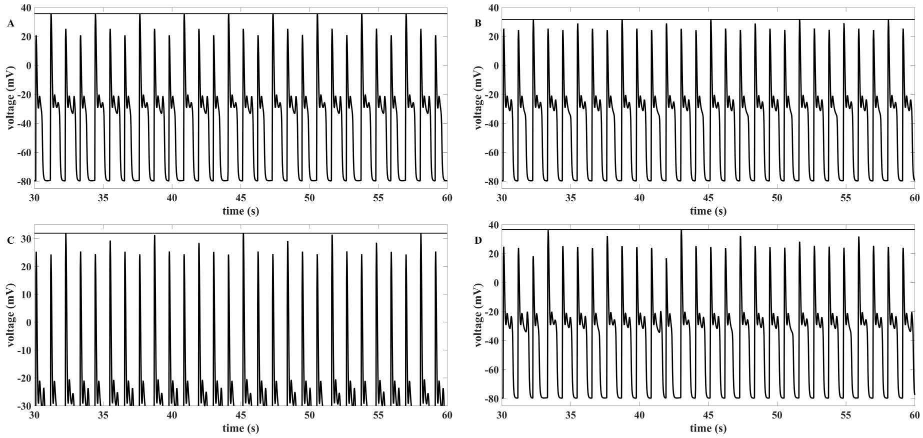

Figure 1: **Period Doubling Route to Chaotic EADs in Model PP.** Evolution of voltage trajectories of model PP (periodically driven at PCL = 1.075s) as the channel conductance G_K is decreased along the period doubling cascade PD1, PD2, PD3, ... (shown in Figure 4PP of the main text). The horizontal line indicates the respective maximum voltage value at display. A) Single period, corresponding to 3PCL, with $G_K = 0.0401$ mS/cm^2 and $G_K > G_{K_1}$ (solid line is hit every three PCL). B) Double period, corresponding to 6PCL, with $G_K = 0.0400301$ mS/cm^2 and $G_{K_1} > G_K > G_{K_2}$ (solid line is hit every six PCL). C) Fourfold period, corresponding to 12PCL, with $G_K = 0.0400301$ mS/cm^2 and $G_{K_2} > G_K > G_{K_3}$ (solid line is hit every twelve PCL) D) Chaotic EADs with $G_K = 0.04$ mS/cm^2 lying beyond the limit of the PD cascade (solid line is hit in a chaotic manner).

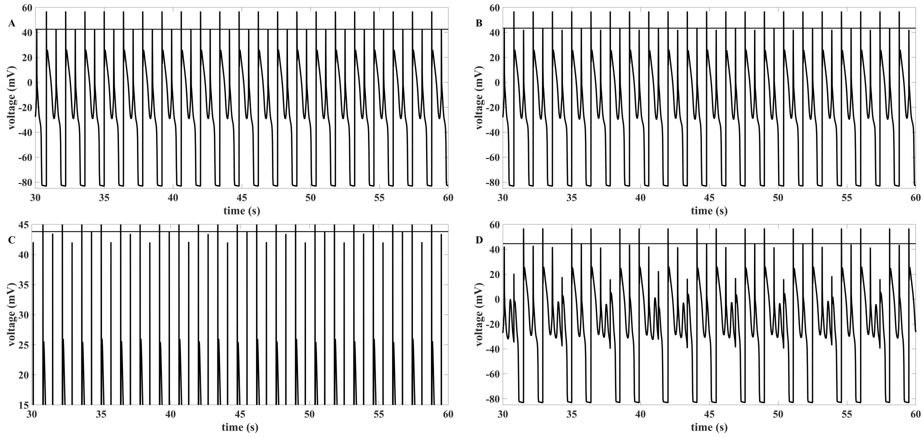

Figure 2: **Period Doubling Route to Chaotic EADs in Model PV.** Evolution of voltage trajectories of model PV (periodically driven at PCL = 0.7s) as the channel conductance G_K is decreased along the period doubling cascade PD1, PD2, PD3, ... (shown in Figure 4PV of the main text). The horizontal line indicates the respective second largest voltage value at display. A) Single period, corresponding to 2PCL, with $G_K = 0.2875$ mS/cm^2 and $G_K > G_{K_1}$ (solid line is hit every two PCL). B) Double period, corresponding to 4PCL, with $G_K = 0.2869$ mS/cm^2 and $G_{K_1} > G_K > G_{K_2}$ (solid line is hit every four PCL). C) Fourfold period, corresponding to 8PCL, with $G_K = 0.286665$ mS/cm^2 and $G_{K_2} > G_K > G_{K_3}$ (solid line is hit every eight PCL) D) Chaotic EADs with $G_K = 0.282$ mS/cm^2 lying beyond the limit of the PD cascade (solid line is hit in a chaotic manner).
